# Real‐world profiles and associated factors of pediatric asthma monitoring

**DOI:** 10.1111/pai.70526

**Published:** 2026-11-09

**Authors:** Eleni Anastasiou, Alexander G. Mathioudakis, Yuichi Adachi, Stefania Arasi, Julijana Asllani, Zeinab A. El‐Sayed, Leonard B. Bacharier, Matteo Bonini, Jose A. Castro‐Rodriguez, Zhimin Chen, Michael Clausen, Timothy Craig, Adnan Custovic, Antoine Deschildre, Zuzana Diamant, Francine M. Ducharme, Philippe Eigenmann, Rasha El‐Owaidy, Wojciech Feleszko, Vincenzo Fierro, Alessandro Fiocchi, Luis Garcia‐Marcos, James E. Gern, Anne E. N. Goh, René Maximiliano Gómez, Maia Gotua, Theresa W. Guilbert, Eckard Hamelmann, Gunilla Hedlin, Elham M. Hossny, Zhanat Ispayeva, Tuomas Jartti, Miloš Jeseňák, Omer Kalayci, Alan Kaplan, Ekaterina Khaleva, Jon Konradsen, Piotr Kuna, Susanne Lau, Peter N. Le Souëf, Robert F. Lemanske, Mika J. Mäkelä, Paolo M. Matricardi, Oleksandr Mazulov, Michael Miligkos, Mário Morais‐Almeida, Clare S. Murray, Karthik Nagaraju, Antonio Nieto Garcia, Zoltan Novak, Emmanouil Paraskakis, Ruby Pawankar, Wanda Phipatanakul, Giorgio Piacentini, Mariëlle W. Pijnenburg, Helena Pite, Paulo M. Pitrez, Petr Pohunek, David B. Price, Alfred Priftanji, Valeria Ramiconi, Daniela Rivero Yeverino, Graham C. Roberts, Aziz Sheikh, Kun‐Ling Shen, Zsolt Szépfalusi, Ioanna Tsiligianni, Mirjana Turkalj, Steve Turner, Tetiana Umanets, Arunas Valiulis, Susanne J. H. Vijverberg, Jiu‐Yao Wang, Tonya A. Winders, Gary W. K. Wong, Paraskevi Xepapadaki, Dong Keon Yon, Su‐Boon Yong, Osman M. Yusuf, Heather J. Zar, Nikolaos G. Papadopoulos

**Affiliations:** ^1^ Allergy Department, Second Pediatric Clinic National and Kapodistrian University of Athens Athens Greece; ^2^ Division of Immunology, Immunity to Infection and Respiratory Medicine, School of Biological Sciences The University of Manchester Manchester UK; ^3^ The North West Lung Centre, Wythenshawe Hospital Manchester University NHS Foundation Trust, Manchester Academic Health Science Centre Manchester UK; ^4^ Pediatric Allergy Center Toyama Red Cross Hospital Toyama Japan; ^5^ Allergy Unit Bambino Gesù Children's Hospital, IRCCS Rome Italy; ^6^ Faculty of Medicine University of Medicine Tirana Albania; ^7^ Pediatric Allergy, Immunology and Rheumatology Unit Children's Hospital, Ain Shams University Cairo Egypt; ^8^ Division of Allergy, Immunology, and Pulmonary Medicine, Department of Pediatrics Monroe Carell Jr Children's Hospital at Vanderbilt University Medical Center Nashville Tennessee USA; ^9^ Department of Public Health and Infectious Diseases Sapienza University of Rome Rome Italy; ^10^ Department of Pediatric Pulmonology, School of Medicine Pontifical Universidad Catolica de Chile Santiago Chile; ^11^ Pulmonology Department Children's Hospital, Zhejiang University School of Medicine Hangzhou China; ^12^ Children's Hospital Landspitali University Hospital Reykjavik Iceland; ^13^ Penn State University Hershey Pennsylvania USA; ^14^ Center of Allergy and Clinical Immunology, Vinmec International Hospital Times City and VinUni Institute of Immunology VinUniversity Hanoi Vietnam; ^15^ National Heart and Lung Institute, Imperial College London London UK; ^16^ Service de Pneumologie et d'Allergologie Pédiatrique Hôpital Jeanne de Flandre, Univ. Lille, CHU de Lille Lille France; ^17^ Department of Clinical Pharmacy and Pharmacology University Medical Center Groningen, University of Groningen Groningen the Netherlands; ^18^ Department of Respiratory Medicine, First Faculty of Medicine Charles University and Thomayer Hospital Prague Czech Republic; ^19^ Department of Pediatrics University of Montreal Montreal Quebec Canada; ^20^ Department of Social and Preventive Medicine University of Montreal Montreal Quebec Canada; ^21^ Department of Women, Children, and Adolescents University Hospital of Geneva Geneva Switzerland; ^22^ Department of Pediatric Pneumonology and Allergy Medical University of Warsaw Children's Hospital Warsaw Poland; ^23^ Pediatric Respiratory and Allergy Units, “Virgen de la Arrixaca” Children's University Clinical Hospital University of Murcia Murcia Spain; ^24^ Departments of Pediatrics and Medicine University of Wisconsin School of Medicine and Public Health Madison Wisconsin USA; ^25^ Department of Paediatrics, Allergy and Respiratory Medicine Service, KK Women's and Children's Hospital SingHealth Singapore Singapore; ^26^ Faculty of Health Sciences Catholic University of Salta Salta Argentina; ^27^ Center for Allergy and Immunology Research (CAIR) Tbilisi Georgia; ^28^ Department of Pediatrics University of Cincinnati and Cincinnati Children's Hospital Medical Center Cincinnati Ohio USA; ^29^ Children's Center Bethel University Hospital OWL, Bielefeld University Bielefeld Germany; ^30^ Department of Women's and Children's Health and Centre for Allergy Research Karolinska Institutet Stockholm Sweden; ^31^ Asfendiyarov Kazakh National Medical University Almaty Kazakhstan; ^32^ Department of Pediatrics Turku University Hospital, University of Turku Turku Finland; ^33^ Department of Paediatrics and Adolescent Medicine, Jessenius Faculty of Medicine in Martin University Hospital, Comenius University in Bratislava Martin Slovakia; ^34^ Pediatric Allergy and Asthma Unit Hacettepe University School of Medicine Ankara Turkey; ^35^ Family Physician Airways Group of Canada University of Toronto Toronto Ontario Canada; ^36^ NIHR Southampton Biomedical Research Centre| University Hospital Southampton NHS Foundation Trust Southampton UK; ^37^ Human Development and Health, Faculty of Medicine University of Southampton Southampton UK; ^38^ Astrid Lindgren's Children's Hospital Karolinska University Hospital Stockholm Sweden; ^39^ Department of Internal Medicine, Asthma and Allergy Medical University of Lodz Lodz Poland; ^40^ Department of Pediatric Respiratory Medicine Immunology and Critical Care Medicine, Charité—Universitätsmedizin Berlin Germany; ^41^ German Center for Child and Adolescent Health (DZKJ) Partner Site Berlin Berlin Germany; ^42^ School of Paediatrics & Child Health, Faculty of Medicine, Dentistry and Health Sciences University of Western Australia Crawley Western Australia Australia; ^43^ Department of Medicine University of Wisconsin School of Medicine and Public Health Madison Wisconsin USA; ^44^ Skin and Allergy Hospital, Helsinki University Hospital (HUS) and University of Helsinki Helsinki Finland; ^45^ Department of Pediatric Pulmonology Immunology and Intensive Care Medicine, Charité—University Medicine Berlin Germany; ^46^ First Pediatric Department National Pirogov Memorial Medical University Vinnytsya Ukraine; ^47^ Allergy Center CUF Descobertas Hospital Lisbon Portugal; ^48^ Division of Immunology, Immunity to Infection and Respiratory Medicine University of Manchester Manchester UK; ^49^ VN Allergy & Asthma Research Centre Chennai India; ^50^ Pediatric Pulmonology and Allergy Unit Hospital Universitari i Politècnic La Fe Valencia Spain; ^51^ Department of Pediatrics University of Szeged Szeged Hungary; ^52^ Department of Paediatrics Heraklion University Hospital, University of Crete Medical School Crete Greece; ^53^ Department of Pediatrics Nippon Medical School Tokyo Japan; ^54^ Boston Children's Hospital, Harvard Medical School Boston Massachusetts USA; ^55^ Pediatric Division, Department of Surgical Sciences, Dentistry, Gynecology and Pediatrics University of Verona Verona Italy; ^56^ Division of Respiratory Medicine and Allergology, Department of Pediatrics University Medical Center Rotterdam, Erasmus MC—Sophia Children's Hospital Rotterdam the Netherlands; ^57^ Immuno‐Allergy Department CUF Tejo Hospital Lisbon Portugal; ^58^ NOVA Medical School Universidade NOVA de Lisboa Lisbon Portugal; ^59^ Hospital Santa Casa de Porto Alegre Porto Alegre Brazil; ^60^ Paediatric Department, 2nd Faculty of Medicine Charles University & Motol and Homolka University Hospital Prague Czech Republic; ^61^ Observational and Pragmatic Research Institute Singapore Singapore; ^62^ Division of Applied Health Sciences, Centre of Academic Primary Care University of Aberdeen Aberdeen UK; ^63^ Department of Allergology and Clinical Immunology UHC Mother Teresa, Medical University Tirana Tirana Albania; ^64^ EFA—European Federation of Allergy and Airways Diseases Patients' Associations Brussels Belgium; ^65^ Department of Allergy and Clinical Immunology| Hospital Universitario de Puebla, Benemérita Universidad Autónoma de Puebla Puebla Mexico; ^66^ NIHR Southampton Biomedical Research Centre University Hospitals Southampton NHS Foundation Trust Southampton UK; ^67^ David Hide Asthma and Allergy Research Centre Isle of Wight UK; ^68^ Nuffield Department of Primary Care Health Sciences University of Oxford Oxford UK; ^69^ Department of Respiratory Medicine Beijing Children's Hospital, Capital Medical University, National Center for Children's Health Beijing China; ^70^ Department of Pediatrics and Adolescent Medicine Medical University of Vienna Vienna Austria; ^71^ Department of Social Medicine, Faculty of Medicine University of Crete Crete Greece; ^72^ Srebrnjak Children's Hospital Zagreb Croatia; ^73^ Catholic University of Croatia Zagreb Croatia; ^74^ Faculty of Medicine J.J. Strossmayer University of Osijek Osijek Croatia; ^75^ Royal Aberdeen Children's Hospital & University of Aberdeen Aberdeen UK; ^76^ SI “Ukrainian Center of Maternity and Childhood of the NAMS of Ukraine” Kyiv Ukraine; ^77^ Institute of Clinical Medicine & Institute of Health Sciences, Medical Faculty Vilnius University Vilnius Lithuania; ^78^ Julius Center for Health Sciences and Primary Care University Medical Center Utrecht, Utrecht University Utrecht the Netherlands; ^79^ China Medical University Children's Hospital, China Medical Universuty Taichung Taiwan; ^80^ GAAPP, Global Allergy & Airways Patient Platform Vienna Austria; ^81^ Department of Paediatrics The Chinese University of Hong Kong, Prince of Wales Hospital Hong Kong China; ^82^ Department of Pediatrics Kyung Hee University Medical Center, Kyung Hee University College of Medicine Seoul South Korea; ^83^ Department of Allergy and Immunology China Medical University Children Hospital Taichung Taiwan; ^84^ The Allergy & Asthma Institute Islamabad Pakistan; ^85^ Department of Pediatrics & Child Health University of Cape Town Cape Town South Africa; ^86^ MRC Unit on Child & Adolescent Health Red Cross War Memorial Children's Hospital, University of Cape Town Cape Town South Africa

**Keywords:** asthma monitoring, clinical practice, home‐monitoring, lung function tests, pediatric asthma, spirometry

## Abstract

**Background:**

Achieving and maintaining pediatric asthma control depends on effective and regular monitoring. While a wide range of monitoring tools are available, how and why clinicians use these in routine practice has not been formally characterized. Here we describe distinct real‐world monitoring patterns among healthcare professionals (HCPs) caring for children with asthma in an international convenience survey, examining the geographic, professional, and institutional factors associated with them.

**Methods:**

Data from the international cross‐sectional Pediatric Asthma in Real Life (PeARL) HCP survey, administered to clinicians with expertise in childhood asthma, were analyzed. Monitoring patterns were derived by Ward agglomerative hierarchical clustering on four standardized domains: clinical evaluation, spirometry, fractional exhaled nitric oxide (FeNO), and home‐monitoring. Independent associations between cluster membership and region, specialty, sector, and level of care were estimated by multinomial logistic regression (MLR).

**Results:**

951 HCPs from 78 countries were included. Four monitoring patterns emerged: Low‐intensity (*n* = 88, 9.3%), lower overall monitoring intensity but moderate clinical assessment; Clinical (*n* = 449, 47.2%), predominantly clinical assessment with moderate spirometry but infrequent FeNO and home monitoring; Clinical & Objective (*n* = 308, 32.4%) with additional regular spirometry with moderate FeNO use and Comprehensive (*n* = 106, 11.1%), also providing home monitoring. In MLR (*n* = 933), region and care level were significantly associated with cluster membership (*p* < .001), and specialty (*p* = .012) and sector (*p* = .039) also contributed independently. The most pronounced gradients involved objective in‐clinic testing: specialists were 3.5‐fold (95% CI 1.68–7.37) more likely than generalists, and tertiary‐care HCPs 3.3‐fold (95% CI 1.99–5.58) more likely than primary‐care HCPs, to belong to the Clinical & Objective pattern, with routine use of objective measures concentrated in Western and Northern European practices. Home monitoring showed no consistent gradient across the measured characteristics.

**Conclusion:**

Real‐world pediatric asthma monitoring follows recognizable patterns, distinguished by the use of objective in‐clinic testing and between‐visit home‐based practices. Nearly half of HCPs relied predominantly on clinical assessment with limited use of FeNO and home‐based monitoring, and only a minority integrated home‐monitoring tools. Objective in‐clinic testing concentrated in specialist services and Western/Northern European practices, while home‐monitoring adoption showed no consistent gradient. These findings identify factors associated with pediatric asthma monitoring and suggest system‐level factors that warrant direct evaluation in dedicated implementation studies.

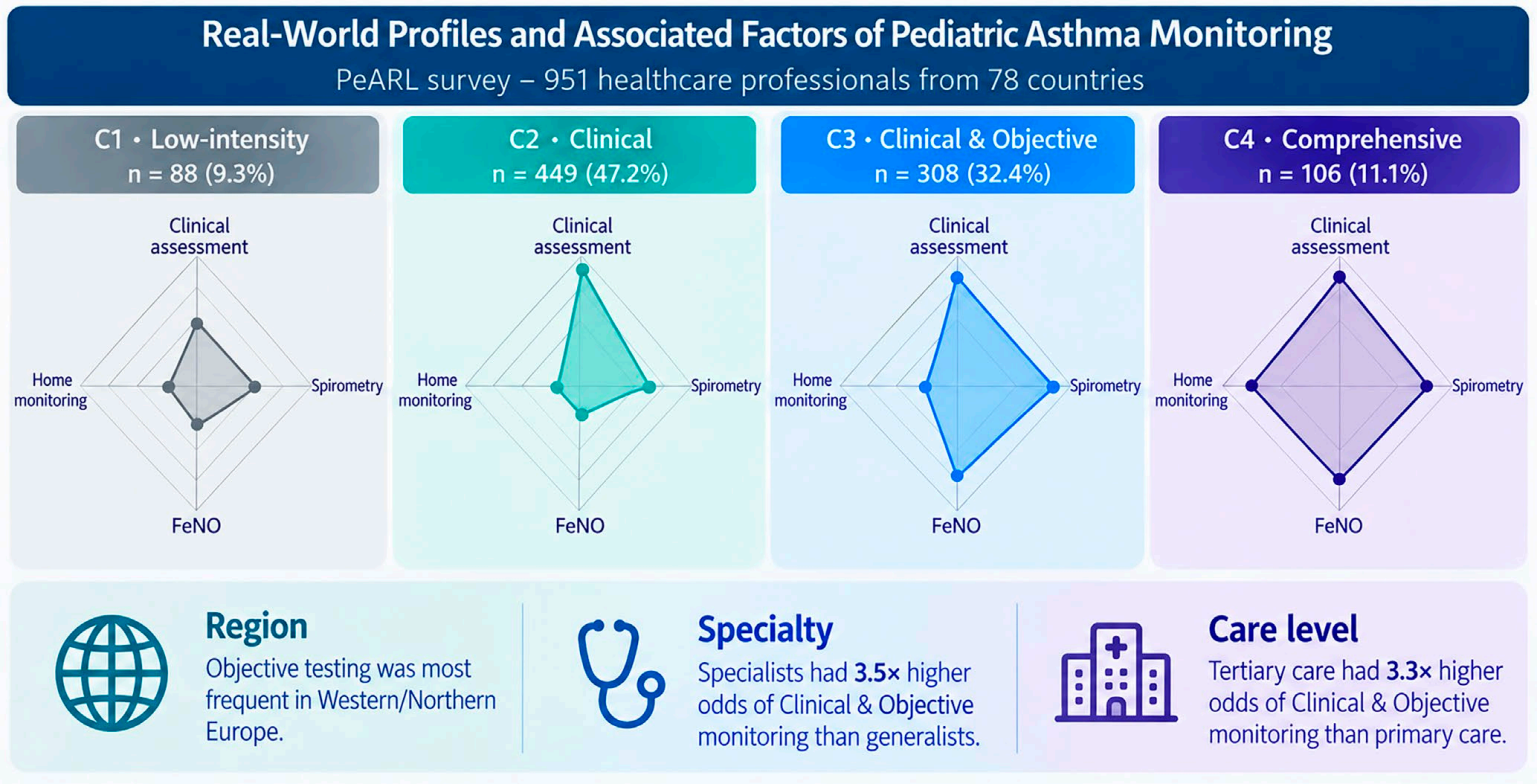

## Graphical abstract

Analysis of the international PeARL survey (951 healthcare professionals, 78 countries) identified four real‐world profiles of pediatric asthma monitoring, from low‐intensity to comprehensive. Objective in‐clinic testing was more likely among specialists and in tertiary care, highlighting variation across clinical settings.

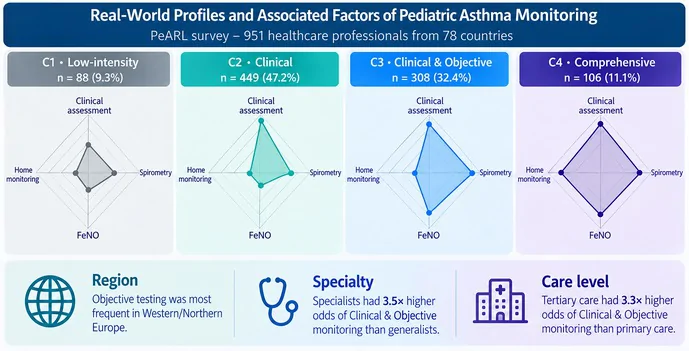

**Abbreviations**
aOR: adjusted odds ratio
ARI: Adjusted Rand Index
CI: confidence interval
EAACI: European Academy of Allergy and Clinical Immunology
FeNO: fractional exhaled nitric oxide
GINA: Global Initiative for Asthma
HCP: healthcare professional
ICS: inhaled corticosteroids
IQR: interquartile range
MLR: multinomial logistic regression
NMI: normalized mutual information
OR: odds ratio
PCP: primary care provider
PeARL: Pediatric Asthma in Real Life
PFT: pulmonary function test
PRO: patient‐reported outcome
REG: Respiratory Effectiveness Group
SD: standard deviation
WCSS: within‐cluster sum of squares
WHO: World Health Organization

**Key message**

Pediatric asthma monitoring varies considerably across clinical settings. Objective tests such as spirometry and FeNO are used more often by specialists, in tertiary care, and in Western and Northern Europe, while home‐monitoring remains uncommon. Improving access to objective testing, particularly in primary care, may help reduce these differences.

## 1 INTRODUCTION

Pediatric asthma remains one of the leading chronic non‐communicable conditions worldwide, associated with substantial morbidity, healthcare resource utilization, and quality of life impairment.^1^, ^2^ Although significant therapeutic advances have been achieved, the economic burden of asthma—driven by both acute exacerbations and ongoing management—remains high and continues to increase.^3^ The primary aim of asthma management is to achieve and maintain disease control, to improve long‐term outcomes,^4^ and, where possible, attain remission.^5^ Central to achieving this goal is regular monitoring.^6^

Asthma monitoring encompasses multiple parameters: clinical assessment and objective measurements, patient‐reported outcomes using validated questionnaires, adherence assessment, allergy evaluation, and digital health tools, among others.^7^, ^8^, ^9^ Although international guidelines recommend regular monitoring, practical guidance on the optimal selection of parameters to prioritize and how often has not been specified until recently.^6^, ^10^, ^11^, ^12^ Recognizing this clinical need,^13^ the Pediatric Asthma in Real Life (PeARL) think tank—an international consortium of healthcare professionals (HCPs), clinical academics, and patient representatives with expertise in pediatric asthma—has generated evidence‐based recommendations for pediatric asthma monitoring in clinical practice.^14^

To better understand real‐world monitoring, PeARL conducted an international survey among HCPs, documenting how children with asthma are monitored across diverse clinical contexts.^15^ The survey indicated widespread implementation of core clinical assessments (history and clinical examination, growth monitoring, spirometry, the Asthma Control Test, adherence/inhalation‐technique evaluation) with no major differences but substantial variability between settings. However, whether this variability reflects distinct monitoring approaches and any factors defining such patterns has not been formally examined. Beyond the item‐level description of monitoring practices reported previously,^15^ a profile‐level analysis of how monitoring components are combined by individual HCPs has not been performed. Therefore, we used the PeARL survey dataset to identify distinct monitoring practice patterns among HCPs and to examine the factors associated with them. Specifically, we aimed to identify distinct monitoring profiles among HCPs, quantify the strength of their association with geographic factors (country/region), professional characteristics (specialty, sector, level of care), and characterize overall features of each.

## 2 METHODS

### 2.1 Study design

This study was performed using the dataset from an international cross‐sectional survey of HCPs caring for children with asthma, the methodology of which has been described in detail previously.^15^ The survey captured the frequency and priority of monitoring domains, tool usage in clinical practice, and demographic details of respondents. The survey was administered via SurveyMonkey between April 12 and September 3, 2021, and disseminated globally through the Respiratory Effectiveness Group and the European Academy of Allergy and Clinical Immunology. Ethics review was not required in line with UK NHS Research Ethics Committee guidance. Participant consent was implied by voluntary completion of the survey. The study followed the American Association for Public Opinion Research (AAPOR) reporting guideline. Responses were anonymous and were considered valid provided the participating HCPs answered at least 1 clinical question. From the records in the original PeARL survey dataset, we used respondents with data for all monitoring variables required for the cluster analysis. The flow diagram is shown in Data SA.

### 2.2 Variables and scales

Four monitoring variables were used for clustering: (1) clinical assessment (asthma‐specific history and clinical examination), (2) spirometry, (3) fractional exhaled nitric oxide (FeNO), and (4) a composite “Home Monitoring” variable combining between‐visit patient‐reported outcomes (ACT/ACQ/CASI/CARATKids) and eHealth applications. These four variables were prespecified to represent the principal monitoring domains defined in the PeARL monitoring framework: symptom assessment, lung function, airway inflammation, and between‐visit home monitoring.

In‐clinic items (clinical assessment, spirometry, FeNO) were rated by the respondent on an ordinal frequency scale and recoded as: Never = 0, Rarely/Once = 1, Occasionally/Sometimes = 2, Almost every visit = 3, Every visit = 4. For the home items, respondents reported the proportion of their patients to whom they recommended each tool; these proportions were recoded onto a 5‐point ordinal scale (0% = 0, 1–25% = 1, 26–50% = 2, 51–75% = 3, >75% = 4) and were averaged into the single Home Monitoring Tools composite. Demographic variables included country of practice, world region, primary specialty (nine self‐reported categories; Table SB1) collapsed for regression into Specialist (Pediatric Allergy, Pediatric Pulmonology, Pediatric Pulmonology & Allergy, Allergy adult‐and‐pediatric), Generalist (Pediatrics, General Practice), and Other (categories with fewer than 20 respondents), level of care (primary, secondary, tertiary), and sector (public, private, mixed).

### 2.3 Cluster analysis

Monitoring profiles were identified by agglomerative hierarchical clustering (Ward's linkage, Euclidean distance) on the four monitoring variables. Variables were standardized (zero mean, unit variance) prior to clustering. Because all four monitoring variables were measured on a common 5‐level ordinal scale (0–4) and standardized prior to clustering, Ward hierarchical clustering with Euclidean distance was considered an appropriate interval‐approximation approach; robustness of the resulting partition was cross‐checked against alternative approaches: the k‐means concordance showed high agreement with the primary partition (ARI = 0.72), Gower‐distance sensitivity analyses (Table SC2) preserved the underlying monitoring‐intensity gradient with substantial cluster‐boundary changes (ARI 0.23–0.27), and bootstrap stability analysis (Figure SC3). The number of clusters was selected by inspecting silhouette scores across 2–10 candidate clusters (*k* = 2–10) and the clinical interpretability of the resulting profiles. To verify that the four clustering variables captured the relevant monitoring information, all non‐included monitoring parameters (adherence, inhaler‐technique assessment, in‐clinic patient‐reported outcomes, peak flow rate, skin‐prick tests and growth monitoring; on the same harmonized ordinal scale) were compared across the four profiles using the Kruskal–Wallis test, with epsilon‐square (*ε* ^2^) as the effect‐size measure (Table SD). Considering that the choice of clustering on four monitoring variables was an analytical assumption, to assess its robustness, we re‐clustered the same standardized four‐variable matrix with k‐means and quantified concordance with the Ward partition using the Adjusted Rand Index (ARI) and Normalized Mutual Information (NMI).

### 2.4 Cluster characterization

For each cluster, mean values of the monitoring variables and demographic composition (country/region, specialty, sector, level of care) were computed. Using the recoded 0–4 scales defined above, in descriptive interpretation, values 0–1 were considered low/infrequent, 2 moderate, and 3–4 frequent. Cluster labels are used throughout as descriptive shorthand for the dominant monitoring pattern of each cluster and are not intended to imply a quality ranking; the exact means of each variable are shown in Figure 1 and Table SD.

**FIGURE 1:**
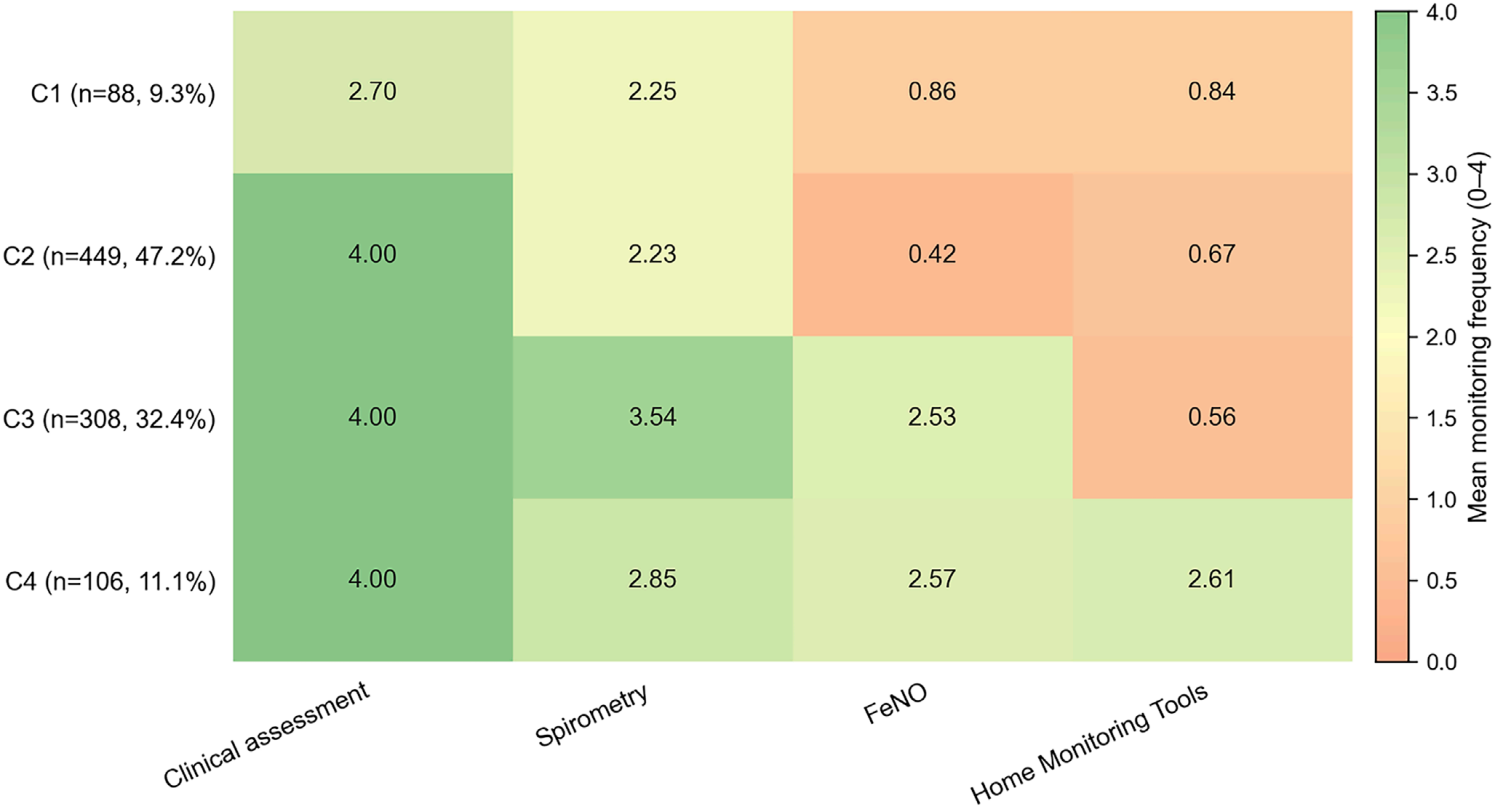
Cluster Profiles—Mean Monitoring frequency per variable (range 0–4; o = never, 4 = every visit) across the four monitoring variables: Clinical assessment, spirometry, FeNO and home‐monitoring tools. C1: Low‐intensity; C2: Clinical; C3: Clinical & Objective; C4: Comprehensive.

### 2.5 Association between cluster membership and demographic factors

Univariate associations were tested with Pearson's chi‐square test and quantified by Cramér's V (<0.10 negligible, 0.10–0.20 weak, 0.20–0.30 moderate, >0.30 strong). Independent effects were estimated with a multinomial logistic regression (outcome: cluster membership, reference C2 Clinical; predictors: Region, Specialty group [Specialist/Generalist/Other], Sector, Care level; reference categories Western Europe, Generalist, Public sector, Primary care; *n* = 933 with complete data), reporting adjusted odds ratios with 95% CIs and Type‐III Wald tests. To account for potential within‐country clustering of respondents' practice patterns, standard errors were computed with a country‐clustered covariance estimator, and a sensitivity analysis was performed excluding respondents from countries with fewer than 5 participants.

### 2.6 Software

All analyses were performed in Python (version 3.13; Python Software Foundation, Wilmington, Delaware, USA) except for the multinomial logistic regression, which was performed in R Software (version 4.5; R foundation for statistical computing, Vienna, Austria).

## 3 RESULTS

### 3.1 Study population

HCPs (*n* = 951) from 78 countries responding to the 2021 PeARL survey were included in this analysis. The sample was geographically diverse, with most respondents from Eastern (27.8%) and Southern Europe (18.3%). All care settings and sectors were represented, with tertiary care (45.7%) and public sector (46.9%) predominance. Clinical specialties included the full spectrum of relevant specialties, both specialist (allergy, pulmonology) and generalist (pediatrics, general practice). Full participant characteristics are provided in Table SB1 and SB2.

### 3.2 Clustering

Hierarchical (Ward) clustering identified four monitoring‐practice patterns (Figure 1). Silhouette analysis across *k* = 2–10 was uniformly low. The numerical maximum occurred at *k* = 6 (0.300), with *k* = 4 (0.278). Detailed inspection of the *k* = 5 and *k* = 6 solutions and bootstrap stability analysis (Figure SC3) showed that the additional profiles emerged as intensity gradients within existing clusters rather than as qualitatively new monitoring approaches. On this basis, *k* = 4 was retained as the most parsimonious partition capturing all qualitatively distinct monitoring approaches supported by the data. Re‐clustering with k‐means showed a highly concordant partition (ARI = 0.72; NMI = 0.70; 88.9% optimal agreement). A further sensitivity analysis separating patient‐reported outcomes and eHealth applications as distinct clustering variables reproduced the same four‐profile structure (best‐alignment agreement 79.5%; ARI = 0.51; NMI = 0.56). A full description of the clusters is provided in Figures SC1–SC3. Cluster 1 (Low‐intensity; *n* = 88, 9.3%) exhibited the lowest overall monitoring intensity, with moderate clinical assessment (mean 2.70) and infrequent use of objective and home‐based tools. Cluster 2 (Clinical; *n* = 449, 47.2%), the largest group, relied predominantly on clinical assessment with only occasional spirometry and infrequent FeNO use. Cluster 3 (Clinical & Objective; *n* = 308, 32.4%) combined clinical assessment with regular use of spirometry and moderate FeNO use, without however between‐visit monitoring. Cluster 4 (Comprehensive; *n* = 106, 11.1%) additionally integrated home‐based monitoring, distinguishing it from C3 predominantly through the home monitoring (Figure 1). All non‐clustering monitoring parameters—including use of peak‐flow, growth measurements, skin‐prick tests, adherence evaluation, inhalation‐technique assessment, and in‐clinic PROs (ACT/ACQ/CASI) showed small effect sizes across clusters (*ε* ^2^ ≤ 0.06, with adherence history at the small‐to‐medium boundary), generally in line with the intensity gradient of the clusters (Table SD).

### 3.3 Association with demographic factors

To analyze the demographic factors associated with the different monitoring patterns, we first performed univariate analysis (Table 1). Region and Specialty showed a moderate association with cluster membership. Sector and Level of care reached statistical significance; however, with weak effect sizes, indicating that institutional factors contribute additional but secondary differentiation between clusters.

**TABLE 1:** Association between cluster membership and demographic factors. [Region: Geographic region of practice, Specialty: Pediatric Allergy, Pediatric Pulmonology, combined Pulmonology & Allergy, General Pediatrics, General Practice, and Allergy (adult‐and‐pediatric). Care level: Primary, secondary, or tertiary. Sector: Public, private, mixed].

Factor	*p*‐value	Cramér's V	Effect Size
Region	<.001	0.231	Moderate
Specialty	<.001	0.207	Moderate
Care level	<.001	0.142	Weak
Sector	<.001	0.151	Weak

Cluster composition varied systematically across HCP groups (Figure 2): the Clinical profile (C2) was substantial in every specialty and region, while respiratory specialists concentrated in the more intensive profiles (C3 and C4) and generalists in C1 and C2. Allergists showed the highest share of the Comprehensive cluster (C4). Care level showed a similar gradient, with the Low‐intensity cluster concentrated in primary care, whereas Comprehensive monitoring was distributed similarly across all care levels. Regional differences were most apparent for the Clinical & Objective profile (C3): 56% of HCPs from Western Europe and 49% from Northern Europe belonged to C3, compared with 29% across other regions. The Clinical profile (C2) was predominant outside Europe (81% in Latin America, 71% in Africa and 64% in Oceania). The complete region × cluster distribution is shown in Table SC1.

**FIGURE 2:**
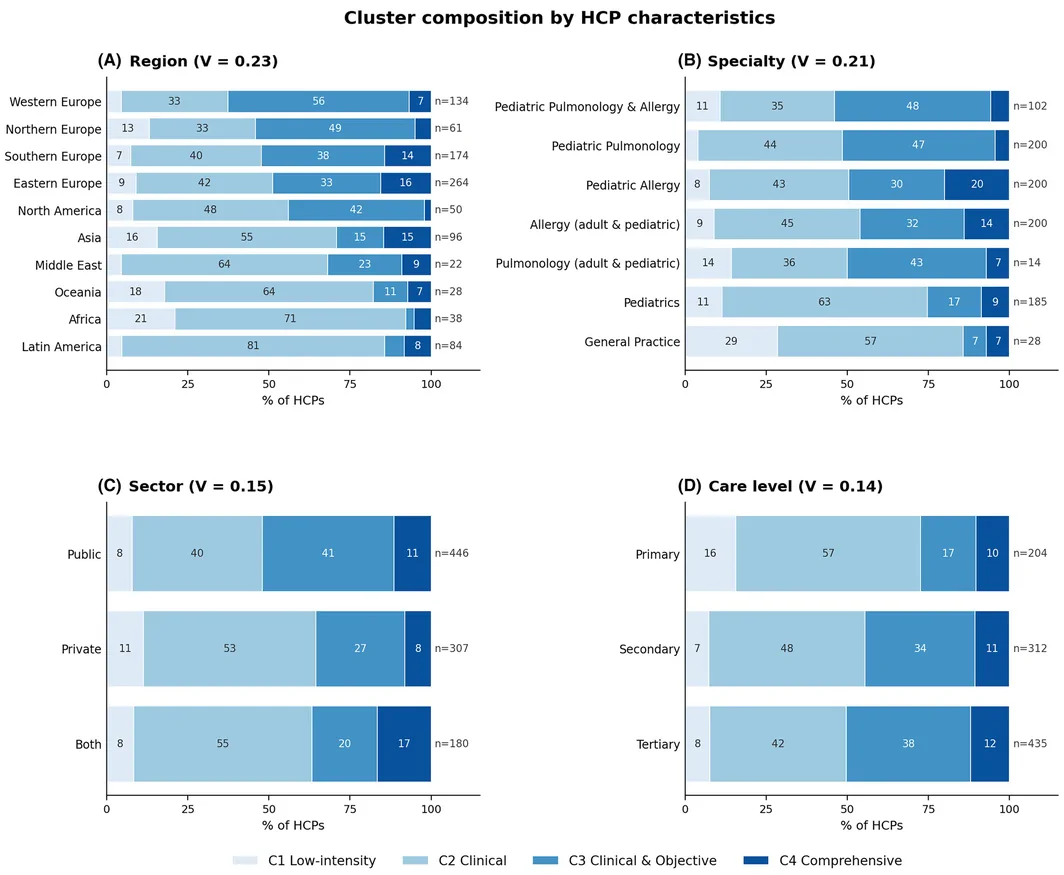
Cluster composition by HCP characteristics. Stacked bars show the percentage of HCPs in each cluster (C1 Low‐intensity, C2 Clinical, C3 Clinical & Objective, C4 Comprehensive) within each group, for (A) Region, (B) Specialty, (C) Sector and (D) Care level. Panel B (Specialty) shows the seven specialty categories with *n* ≥ 20 respondents. Pediatric Immunology (*n* = 9) and Other (*n* = 13) are omitted from this panel due to insufficient sample size for cluster‐level visualization; both categories were retained in the multinomial regression under the ’Other’ grouping.

### 3.4 Independent associations

To assess which demographic and institutional factors remained independently associated with cluster membership after mutual adjustment, a multinomial logistic regression was fitted with the Clinical cluster (C2, the largest group) as the reference outcome and Western Europe, Generalist, Public sector, and Primary care as reference levels (*n* = 933 with complete covariate information. 18 HCPs (1.9%) with missing Sector data were excluded from this model only, while all descriptive analyses retained the full cohort of *n* = 951). Adjusted odds ratios were estimated jointly for all three non‐reference clusters (C1, C3, and C4) versus C2.

All four factors remained independently associated with cluster membership. Adjusted odds ratios for each cluster contrast are presented in Figure 3. The strongest and most consistent associations emerged for the Clinical & Objective cluster. Specialists were 3.5‐fold more likely than generalists, and tertiary‐care HCPs were 3.3‐fold more likely than primary‐care HCPs to be in C3, with secondary care intermediate. Regional contrasts were pronounced: compared with Western Europe (reference region), the adjusted odds of being in C3 were markedly lower in Latin America, Africa, Asia, the Middle East, and Oceania. Differences within Europe and between North America and Western Europe were not statistically significant. A sensitivity analysis excluding countries with fewer than 5 participants yielded near‐identical estimates. The Low‐intensity cluster showed limited demographic structure: secondary‐care HCPs were less likely than primary‐care HCPs to be in C1, with no other significant predictors.

**FIGURE 3:**
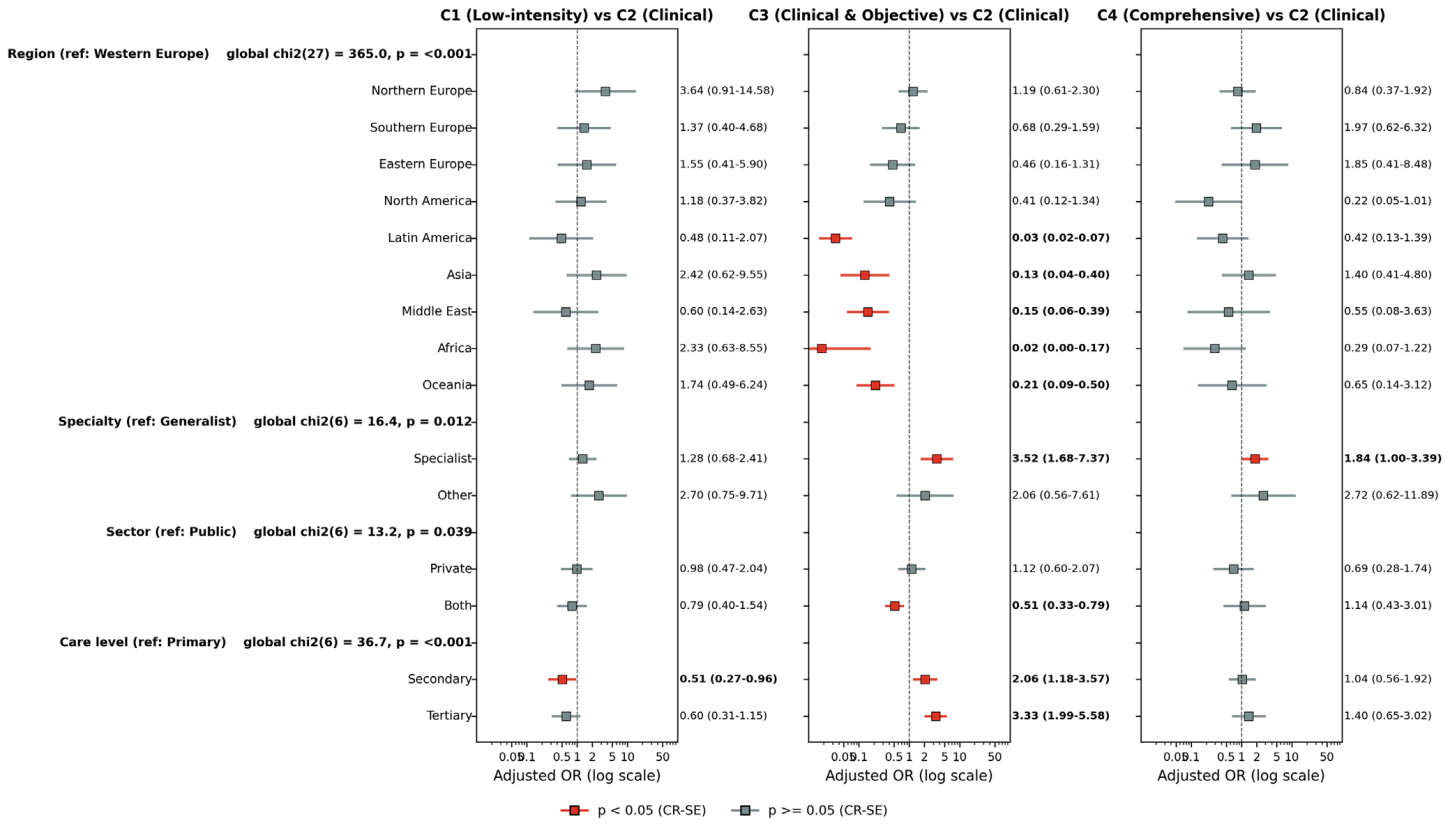
Adjusted odds ratios (95% CI) for cluster membership from a multinomial logistic regression with country‐clustered robust standard errors (*n* = 933 HCPs from 78 countries). Reference outcome: C2 (Clinical). Reference predictors: Western Europe, Generalist, Public sector, Primary care. Headers show global type III Wald tests for each predictor block; red intervals indicate *p* < .05, gray intervals *p* ≥ .05.*.

The Comprehensive cluster (C4) was not significantly associated with any region, sector, or care level after adjustment, indicating that no clear association was detected with the limited set of structural variables available in this survey (region, sector, care level, specialty).

## 4 DISCUSSION

In this analysis of the international PeARL pediatric asthma monitoring survey conducted in 2021, we identified four real‐world monitoring profiles and examined how region, sector, specialty, and level of care relate to monitoring choices. The PeARL Think Tank has previously issued recommendations for pediatric asthma monitoring.^14^

The four profiles describe a clear gradient of monitoring intensity, from minimal engagement through a predominantly clinical approach, to the addition of objective measurements and, finally, a fully integrated profile that also includes patient‐side home monitoring. The fact that nearly half of HCPs monitor pediatric asthma primarily through clinical assessment, with only occasional use of objective/between‐visit tools, is a striking finding. Symptoms alone are known to underestimate ongoing airway inflammation and poor control^16^ and monitoring strategies built around objective tools have been shown to reduce exacerbations in children compared with symptom‐based follow‐up^17^ while FeNO can additionally predict asthma relapse in children with clinical remission.^18^ Distinct trajectories of airflow limitation are largely set in childhood and track into adult chronic obstruction^19^, ^20^ and a substantial share of adult asthma and COPD is now traced back to impaired early life lung development.^21^ Biomarkers such as FeNO are increasingly central to pediatric asthma assessment^22^ and to phenotype‐targeted treatment decisions in children.^23^ Given these considerations, universal lung function assessment in asthma is a reasonable starting point, since the evidence base and infrastructure are more developed. The lessons learned could then inform how objective lung‐function assessment can be gradually integrated into broader pediatric practice.

Across specialty, the cleanest signal was the dichotomy between specialists and generalists in the use of objective tools. We show that specialists and tertiary‐care HCPs are more likely to incorporate objective tools into their clinical practice than primary‐care HCPs and generalists. Studies have shown that pediatricians and family physicians use objective measures far less often than subspecialists, despite recognizing their value.^24^, ^25^, ^26^, ^27^, ^28^, ^29^ Primary care barriers have been described in the past, including limited equipment, lack of formal training in test interpretation, time pressure during short visits, age‐related feasibility issues in preschool children where conventional spirometry is rarely cooperative and oscillometry is not widely available, and unclear reimbursement.^30^, ^31^ On the other hand, it is well established that children monitored by respiratory/allergy specialists achieve better symptom control, receive controller therapy more consistently and are hospitalized less often than those followed exclusively in primary care.^32^, ^33^ Targeted future educational interventions for generalists and primary‐care HCPs together with system‐level support (equipment access, dedicated visit time, better‐defined reimbursement policies) could narrow this difference and support a more uniform standard of pediatric asthma monitoring across care settings. Clear referral pathways to specialized care may also further enhance this effort.

Childhood asthma prevalence and severity vary widely across regions^34^ and international comparisons of asthma control rates have linked these gradients to differences in healthcare system organization, reimbursement, and access to equipment rather than to clinician choices.^35^ International guidelines emphasize regular monitoring and the use of objective control assessment^36^ and adult asthma has been shown to vary widely across countries in burden and management.^37^ Our findings extend this picture: routine use of objective tools was substantially lower in Africa, Asia, Latin America, the Middle East, and Oceania compared with European practices. This difference remained after mutual adjustment for specialty, sector, and care level (with standard errors clustered by country) and country level and plausibly reflects differences in monitoring practice across health systems. Home‐monitoring tools were the least frequently adopted monitoring tool, with just one in ten HCPs including it in their practice. The literature of the past decade underlines the value of such tools for asthma control and adherence.^38^ In pediatric populations, however, dedicated programmes evaluating home‐monitoring and mHealth interventions have been proposed and are under active investigation^39^, ^40^ though real‐world data are still limited. Recent analyses point to structural rather than perceptual barriers like clinician training, data integration into electronic records, reimbursement, and the absence of robust cost‐effectiveness evidence.^41^, ^42^ Additional pediatric‐specific obstacles may include the limited availability of child‐oriented digital tools validated in local languages and the lack of dedicated clinical resources to review patient‐generated data between visits and translate them into management decisions.^43^, ^44^ Our findings are consistent with this evidence base by showing that HCPs themselves consider these tools valuable but only a small minority of clinicians use them.

### 4.1 Strengths and limitations

A major strength of this analysis is that the PeARL survey enrolled a large, international, and multidisciplinary sample of clinicians caring for children with asthma, providing a unique opportunity to examine real‐world monitoring practices beyond the boundaries of any single health system. The use of unsupervised clustering, with a complete‐case multinomial modeling, allowed monitoring patterns to emerge from the data themselves rather than being imposed by guideline categories.

Several limitations must nevertheless be acknowledged. The survey was cross‐sectional and based on self‐reported behavior, which may overstate adherence to objective monitoring and cannot establish causal relationships between structural factors and practice patterns. Recruitment was through professional networks rather than population sampling, so the cohort may not be representative of pediatric asthma care globally; in particular, primary‐care clinicians and low‐resource settings are under‐represented. Comparison with excluded respondents was possible only for country of practice and showed a similar overall European proportion, although the detailed regional distribution differed significantly. Although the sample spanned 78 countries, individual regions contained few respondents from some areas, and the regional categories inevitably mask within‐region heterogeneity. The regional gradient may reflect factors not measured in the present survey: patient‐level outcomes, on‐site equipment availability (spirometers, FeNO analyzers, eHealth platforms), reimbursement, or time constraints directly; structural explanations therefore remain inferential and should be tested in dedicated implementation studies. Some of the observed variation, especially the predominance of the Clinical profile in primary care, may reflect appropriate alignment to mild asthma cases rather than true under‐monitoring. Bootstrap resampling showed reasonable stability of the Low‐intensity profile (Jaccard 0.62) but modest stability of the boundaries between the three higher‐intensity profiles (Jaccard 0.37–0.45), consistent with the low silhouette values; individual respondent assignment should therefore be interpreted with caution. A sensitivity analysis separating patient‐reported outcomes and eHealth applications as distinct clustering variables reproduced the same four‐profile structure but with some redistribution between the Clinical and Comprehensive profiles, indicating that the exact cluster boundaries—though not the overall structure—depend to some extent on the composite construction. The survey was administered in 2021 and the findings represent monitoring practices at that time; digital and home‐monitoring tools have evolved considerably since then, and the patterns reported here should be interpreted as a 2021 snapshot rather than as current practice. The four monitoring variables were treated as approximately interval‐scaled after standardization; sensitivity analyses using Gower distance (an ordinal‐appropriate metric) preserved the same monitoring‐intensity gradient but yielded partly different cluster boundaries, indicating that specific cluster labels are metric‐dependent while the broader pattern structure is stable. Finally, the analysis was restricted to four monitoring components and a single composite home‐monitoring measure; richer instruments and longitudinal designs will be needed to characterize how monitoring patterns evolve over time and translate into clinical benefit.

### 4.2 Clinical implications and future directions

Three key practical messages emerge from our findings. First, pediatric asthma monitoring is not a single behavior but a small set of recognizable patterns, separated mainly by the use of objective in‐clinic testing and the integration of home‐based tools; quality‐improvement efforts that treat “monitoring” as a uniform activity are likely to miss this structure and should instead be tailored to the dominant pattern in a given setting. Second, the observed links identify hypotheses about potentially modifiable factors—including but not limited to equipment access, training and reimbursement—none of which were directly measured in this survey and all of which require direct evaluation. These include coverage of objective tests, equipment provision in primary care (e.g. portable spirometers with built‐in quality grading and automated feedback on suboptimal maneuvers), training pathways for generalists, dedicated personnel for monitoring education (hands‐on/telehealth coaching), and integration of validated digital tools into routine workflows. Third, the persistent under‐use of home and digital monitoring identifies a clear opportunity area for structured between‐visit programmes supported by digital tools. The present analysis describes clinician reporting and cannot directly verify what is delivered at the bedside or how this relates to patient outcomes. To address this, a follow‐up PeARL initiative is currently underway in which patient‐level data are gathered. Linking patient‐level data to the patterns defined here will allow us to test whether the structural gradients observed translate into measurable differences in asthma control and enable context‐tailored strategies to improve it.

## AUTHOR CONTRIBUTIONS

**Eleni Anastasiou:** Methodology; software; data curation; writing – original draft; writing – review and editing; visualization. **Alexander G. Mathioudakis:** Methodology; writing – review and editing; data curation; visualization. **Yuichi Adachi:** Writing – review and editing. **Stefania Arasi:** Writing – review and editing. **Julijana Asllani:** Writing – review and editing. **Zeinab A. El‐Sayed:** Writing – review and editing. **Leonard B. Bacharier:** Writing – review and editing. **Matteo Bonini:** Writing – review and editing. **Jose A. Castro‐Rodriguez:** Writing – review and editing. **Zhimin Chen:** Writing – review and editing. **Michael Clausen:** Writing – review and editing. **Timothy Craig:** Writing – review and editing. **Adnan Custovic:** Writing – review and editing. **Antoine Deschildre:** Writing – review and editing. **Zuzana Diamant:** Writing – review and editing. **Francine M. Ducharme:** Writing – review and editing. **Philippe Eigenmann:** Writing – review and editing. **Rasha El‐Owaidy:** Writing – review and editing. **Wojciech Feleszko:** Writing – review and editing. **Vincenzo Fierro:** Writing – review and editing. **Alessandro Fiocchi:** Writing – review and editing. **Luis Garcia‐Marcos:** Writing – review and editing. **James E. Gern:** Writing – review and editing. **Anne E. N. Goh:** Writing – review and editing. **René Maximiliano Gómez:** Writing – review and editing. **Maia Gotua:** Writing – review and editing. **Theresa W. Guilbert:** Writing – review and editing. **Eckard Hamelmann:** Writing – review and editing. **Gunilla Hedlin:** Writing – review and editing. **Elham M. Hossny:** Writing – review and editing. **Zhanat Ispayeva:** Writing – review and editing. **Tuomas Jartti:** Writing – review and editing. **Miloš Jeseňák:** Writing – review and editing. **Omer Kalayci:** Writing – review and editing. **Alan Kaplan:** Writing – review and editing. **Ekaterina Khaleva:** Writing – review and editing. **Jon Konradsen:** Writing – review and editing. **Piotr Kuna:** Writing – review and editing. **Susanne Lau:** Writing – review and editing. **Peter N. Le Souëf:** Writing – review and editing. **Robert F. Lemanske:** Writing – review and editing. **Mika J. Mäkelä:** Writing – review and editing. **Paolo M. Matricardi:** Writing – review and editing. **Oleksandr Mazulov:** Writing – review and editing. **Michael Miligkos:** Writing – review and editing. **Mário Morais‐Almeida:** Writing – review and editing. **Clare S. Murray:** Writing – review and editing. **Karthik Nagaraju:** Writing – review and editing. **Antonio Nieto Garcia:** Writing – review and editing. **Zoltan Novak:** Writing – review and editing. **Emmanouil Paraskakis:** Writing – review and editing. **Ruby Pawankar:** Writing – review and editing. **Wanda Phipatanakul:** Writing – review and editing. **Giorgio Piacentini:** Writing – review and editing. **Mariëlle W. Pijnenburg:** Writing – review and editing. **Helena Pite:** Writing – review and editing. **Paulo M. Pitrez:** Writing – review and editing. **Petr Pohunek:** Writing – review and editing. **David B. Price:** Writing – review and editing. **Alfred Priftanji:** Writing – review and editing. **Valeria Ramiconi:** Writing – review and editing. **Daniela Rivero Yeverino:** Writing – review and editing. **Graham C. Roberts:** Writing – review and editing. **Aziz Sheikh:** Writing – review and editing. **Kun‐Ling Shen:** Writing – review and editing. **Zsolt Szépfalusi:** Writing – review and editing. **Ioanna Tsiligianni:** Writing – review and editing. **Mirjana Turkalj:** Writing – review and editing. **Steve Turner:** Writing – review and editing. **Tetiana Umanets:** Writing – review and editing. **Arunas Valiulis:** Writing – review and editing. **Susanne J. H. Vijverberg:** Writing – review and editing. **Jiu‐Yao Wang:** Writing – review and editing. **Tonya A. Winders:** Writing – review and editing. **Gary W. K. Wong:** Writing – review and editing. **Paraskevi Xepapadaki:** Writing – review and editing. **Dong Keon Yon:** Writing – review and editing. **Su‐Boon Yong:** Writing – review and editing. **Osman M. Yusuf:** Writing – review and editing. **Heather J. Zar:** Writing – review and editing. **Nikolaos G. Papadopoulos:** Conceptualization; methodology; data curation; supervision; writing – original draft; writing – review and editing; project administration.

## FUNDING INFORMATION

The original Pediatric Asthma in Real Life (PeARL) monitoring survey was supported by the Respiratory Effectiveness Group. This study did not receive further funding.

## CONFLICT OF INTEREST STATEMENT

N.G.P.: Nestlé Nutrition Institute, Numil Hellas SA, GSK, HAL Allergy Holding B.V., Regeneron Pharmaceuticals, Inc., Integris Pharma SA, Berlin—Chemie AG, DBV Technologies SA, Vibrant America, Hyproca Nutrition USA INC, Danone Trading Medical B.V., Med Maps srl, Danone Global Research & Innovation Center B.V., VIAN S.A., ELPEN Pharmaceutical Co. Inc.; A.C.: Custovic reports personal fees from Amgen, personal fees from La Roche‐Posay, personal fees from Reacta Healthcare, personal fees from Stallergenes Greer, personal fees from OM Pharma, personal fees from Allergopharma, personal fees from Thermo Fisher Scientific, outside the submitted work; S.A.: has participated as an advisory board mem‐ber, and/or consultant, and/or speaker/chair at scientific meetings for Aimmune, DBV, Ferrero, Mabylon, Novartis, Stallergenes Greer, ThermoFisher Scientific, and RAPT‐GSK outside the submitted work. Research support was provided to her institution by the Italian Minister of Health and Italian Minister of Education. All of these outside the submitted work.; MB: Research grants, consultancy and lecture fees from Abbvie, AZ, Celltrion, Chiesi, Lallemand, Lusofarmaco, Menarini, Niox, Pfizer, Regeneron, Sanofi; L.B.B.: member of the GINA Science Committee; reports grants from NIH/NIAID/NHLBI, personal fees from GlaxoSmithKline, Genentech/Novartis, AstraZeneca, Avillion, WebMD/Medscape, Sanofi/Regeneron, Circassia, OM Pharma, Apogee, Excellergy, and Kinaset, for DSMB fees from AstraZeneca, DBV Technologies, Aravax, AbbVie, Horizon, and Vertex; and royalties from Elsevier and Up‐To‐Date outside the submitted work. T.C.: conflicts are research conflicts with ADARx, Argo, BioMarin, Kalvista, Pharvaris, GSK, CSL Behring, Takeda, Ionis, Intellia, Astria, Pfizer, Regeneron, Sanofi and Imbrex; speaking conflicts for Takeda, CSL Behring, Kalvista, Ionis, and Grifols; consultant conflicts for Argo, BioMarin, Intellia, CSL Behring, Takeda, BioMarin, Ionis, Astria, Kalvista, CSL Behring, and Biocryst. He also has Center designations from the International Hereditary Angioedema Association and Alpha‐1 Foundation and is a member of the Medical Advisory Board for the HAE‐A; A.D.: received speaker/consulting fees from Aimmune Therapeutics, ALK, AstraZeneca, Celltrion, DBV Technologies, GSK, Novartis, Regeneron Pharmaceuticals Inc., Sanofi, Stallergenes Greer, and Viatris, outside the submitted work; FMD has received: investigator‐initiated research funds from Canadian Institutes of Health Research, Concours Projet Intersectoriel of the Réseau de recherche en santé respiratoire; Fonds d'innovation—Propulsion Santé, of the Ministère de la santé et des services sociaux (MSSS) in partnership with AstraZeneca, Institut national de santé publique du Québec (INSPQ), Fonds de la recherche en santé du Québec (FRSQ), Réseau de recherche en santé CardioMétabolique, Diabète et Obésité (CMDO), et G3 de la Francophonie; H.S.J.: honorarium for consultancy work or advisory board from Canadian Institute of Health Research; honorarium as an invited speaker from Association des Médecins omnipraticiens du Richelieu Saint‐Laurent, Association des médecins francophones du Canada, Astra Zeneca, Direction du Développement professionnel continu (DPC) de la faculté de médecine de l'Université de Montréal, Fédération des Médecins Omnipraticiens du Québec, Sanofi‐Régéneron, and Thorasys Inc.; JEG has received consulting fees from Gilead Sciences and Arrowhead Pharmaceuticals.; R.M.G.: Sanofi & GSK speaker honoraria for asthma; T. W. Guilbert has received personal fees from Advent, AiCME, American Board of Pediatrics, American Thoracic Society, Amgen, AstraZeneca, Genentech, OM Pharma, PlatformQ Health, Regeneron Pharmaceuticals Inc., Sanofi, and Teva; has received grants from Amgen, AstraZeneca, GSK, NIH, OM Pharma, Regeneron Pharmaceuticals Inc., and Sanofi; and has received royalties from UpToDate; MJ reports speaker's fee from ALK, Stallergenes‐Greer, AstraZeneca, Chiesi, BerlinChemie Menarini, Zentiva, Viatris, Novartis, Takeda; served as a member of Advisory boards for Stallergenes‐Greer, Chiesi, AstraZeneca, ALK, Novartis, Takeda and received travel grants from Takeda, BerlinChemie Menarini, ALK, Stallergenes‐Greer and Novartis.; Helena Pité reports fees for lectures or advisory board participation (last 5 years) from AstraZeneca, Chiesi, FAES Farma, GSK, JABA Recordati, Leti, Medinfar, Menarini, OM‐Pharma, Organon, Tecnifar, Viatris; clinical trials paid to her Institution from Novartis and AstraZeneca; CSM reports personal fees from GSK, Sanofi, Astra Zeneca; Grants from NIHR, MRC, Innovate UK, Asthma and Lung UK, Jon Moulton Charity trust, GSK and Thermo Fisher Scientific; AK Advisory Board or Speakers bureau for Amgen, ALK, AZ, GSK, Idorsia, Merck, Pfizer, Sanofi, Trudel and Valeo; S.L.: is honoraria for advisory boards by Amgen, AstraZeneca, GSK, and Sanofi for biologics; S.L.: has received honoraria for advisory boards by AstraZeneca, GSK and Sanofi for biologics; E.P.: GSK, MSD, Pfizer, Innovis, Astrazeneca; W.P.: Consulting sanofi, regeneron, novartis, genentech, Astra Zeneca, GSK, asthma therapeutics; P.P.: Advisory board and lecturer activity AstraZeneca, GlaxoSmithKline; David B. Price has advisory board membership with AstraZeneca, Boehringer Ingelheim, Chiesi, GlaxoSmithKline, OM Pharma, Novartis, Viatris, Teva Pharmaceuticals; consultancy agreements with AstraZeneca, Boehringer Ingelheim, Chiesi, GlaxoSmithKline, Novartis, Viatris, Teva Pharmaceuticals; grants and unrestricted funding for investigator‐initiated studies (conducted through Observational and Pragmatic Research Institute Pte Ltd) from AstraZeneca, Chiesi, Viatris, Novartis, Regeneron Pharmaceuticals, Sanofi Genzyme, and UK National Health Service; payment for lectures/speaking engagements from AstraZeneca, Boehringer Ingelheim, Chiesi, Cipla, Inside Practice, GlaxoSmithKline, Med Maps, Medscape, Viatris, Novartis, Regeneron Pharmaceuticals and Sanofi Genzyme, Teva Pharmaceuticals; payment for travel/accommodation/meeting expenses from AstraZeneca, Boehringer Ingelheim, Novartis, Medscape, Teva Pharmaceuticals; owns 74% of the social enterprise Optimum Patient Care Ltd. (Australia and UK) and 92.61% of Observational and Pragmatic Research Institute Pte Ltd. (Singapore); is peer reviewer for grant committees of the UK Efficacy and Mechanism Evaluation Programme, and Health Technology Assessment; and he was an expert witness for GlaxoSmithKline; AD is a member of the steering committee of the European Respiratory Society Clinical Research Collaboration SPACE, which has been or is funded by Novartis, AstraZeneca, Sanofi, and Celltrion; G.W.K.W.: i have no conflicts relevant to this manuscript but am a paid speaker and advisor for unbranded disease awareness and education for AstraZeneca, Chiesi, Eli Lilly, GSK, Novartis, Sanofi Regeneron and TEVA; MWP is chair of the steering committee of the European Respiratory Society Clinical Research Collaboration SPACE, which has been or is funded by Novartis, AstraZeneca, Sanofi, and Celltrion. She received consultancy fees from Sanofi (paid to her institution), she is a member of a DMC for Novartis (paid to her institution); P.K.: Honoraria for lectures were received from AstraZeneca, Adamed, Berlin‐Chemie Menarini, GSK, FAES, Chiesi, Polpharma, Teva, and Glenmark; Y.A.: Speaker fees were received from Otsuka Pharmaceutical Co., Viatris, Muranaka Medical Instrument Co., AstraZeneca, GSK, AbbVie, Torii Pharmaceutical Co., and Novartis; P.M.P.: consultancy and lecture fees from AZ, Sanofi, GSK, Boehringer Ingelheim, and Aché. G.P.: received speaker/consulting fees from GSK, Recordati, Chiesi, Noos, Angelini, Regeneron Pharmaceuticals, Sanofi, Astra Zeneca, outside the submitted work;The other authors does not report any conflict of interest related to this work.

## ETHICS STATEMENT

As detailed in the Methods, this healthcare‐professional survey did not require formal ethics review under UK NHS Research Ethics Committee guidance for such survey designs. All participants provided informed consent through voluntary, anonymous participation.

## CONSENT

All authors have given their consent for publication.

## Supporting information


Data S1.
**Figure SA1**. Flow diagram.
**Table SB1**. HCPs Characteristics.
**Table SB2**. Countries.
**Figure SC1**. Silhouette analysis—Ward Hierarchical clustering on 4 monitoring variables.
**Figure SC2**. Algorithm sensitivity: Ward vs. k‐means partitions. Cross‐tabulation of cluster assignments produced by Ward agglomerative clustering (rows) and k‐means clustering on the same standardized four‐variable matrix (*n* = 951; k = 4).
**Figure SC3**. Cluster profiles across candidate solutions (*k* = 2 to *k* = 6). C = Cluster.
**Table SC1**. Region‐by‐cluster contingency table for the analytical cohort (*n* = 933 HCPs with complete data). Cell entries are number of respondents and (row percentage). Cluster labels: C1 = Low‐intensity; C2 = Clinical (reference); C3 = Clinical & Objective; C4 = Comprehensive.
**Table SC2**. Gower‐distance sensitivity analysis.
**Table SD**. All monitoring parameters in PeARL survey.

## Data Availability

Data available upon request.
