# Supplementary material for: Real‐world profiles and associated factors of pediatric asthma monitoring
Source: Pediatr Allergy Immunol. 2026 Nov 9;37(11):e70526. doi: 10.1111/pai.70526 (PMC13746309; doi:10.1111/pai.70526)
Supplement: Supplementary file 1 [file PAI-37-e70526-s001.docx]

Supplement A

Supplement Figure A. Flow diagram

**Participant flow diagram**

**Original PeARL international survey**

n = 1,757

**↓**

| **Complete demographic data**  (country, region, specialty,  sector, level of care)  n = 1,255 | **Excluded: n = 502**  Missing demographic data |
| --- | --- |

**↓**

| **Complete data on the 4**  monitoring variables required  for the cluster analysis  n = 951 | **Excluded: n = 304**  Missing data on ≥ 1 of the  4 monitoring variables |
| --- | --- |

**↓**

**Analytical cohort**

n = 951 HCPs from 78 countries

**↓	↓**

| **Hierarchical clustering**  (Ward / Euclidean,  4 standardised variables)  k = 4 monitoring profiles | **Demographic associations**  χ² + Cramér's V  Multinomial logistic regression  (n = 933 complete cases) |
| --- | --- |

Supplement B

Supplement Table B1. HCPs Characteristics

| **Characteristic** | **n** | **%** |
| --- | --- | --- |
| **Region** |  |  |
| Eastern Europe | 264 | 27.8% |
| Southern Europe | 174 | 18.3% |
| Western Europe | 134 | 14.1% |
| Asia | 96 | 10.1% |
| Latin America | 84 | 8.8% |
| Northern Europe | 61 | 6.4% |
| North America | 50 | 5.3% |
| Africa | 38 | 4.0% |
| Oceania | 28 | 2.9% |
| Middle East | 22 | 2.3% |
| **Specialty** |  |  |
| Pediatric Allergy | 200 | 21.0% |
| Pediatric Pulmonology | 200 | 21.0% |
| Allergy (adult & pediatric) | 200 | 21.0% |
| Pediatrics | 185 | 19.5% |
| Pediatric Pulmonology & Allergy | 102 | 10.7% |
| General Practice | 28 | 2.9% |
| Pulmonology (adult & pediatric) | 14 | 1.5% |
| Other | 13 | 1.4% |
| Pediatric Immunology | 9 | 0.9% |
| **Level of care** |  |  |
| Tertiary | 435 | 45.7% |
| Secondary | 312 | 32.8% |
| Primary | 204 | 21.5% |
| **Sector** |  |  |
| Public | 446 | 46.9% |
| Private | 307 | 32.3% |
| Both | 180 | 18.9% |
| Not reported | 18 | 1.9% |

Supplement Table B2. Countries

| **Country** | **n** | **%** | **Country** | **n** | **%** |
| --- | --- | --- | --- | --- | --- |
| Greece | 76 | 8.0% | Croatia | 4 | 0.4% |
| Spain | 63 | 6.6% | Hong Kong | 4 | 0.4% |
| Hungary | 56 | 5.9% | Iceland | 4 | 0.4% |
| Russian Federation | 45 | 4.7% | Kenya | 4 | 0.4% |
| Ukraine | 38 | 4.0% | Peru | 4 | 0.4% |
| United Kingdom | 38 | 4.0% | Denmark | 3 | 0.3% |
| United States | 36 | 3.8% | Malaysia | 3 | 0.3% |
| France | 33 | 3.5% | Norway | 3 | 0.3% |
| Japan | 31 | 3.3% | Philippines | 3 | 0.3% |
| India | 30 | 3.2% | Zimbabwe | 3 | 0.3% |
| Germany | 28 | 2.9% | Belgium | 2 | 0.2% |
| Mexico | 28 | 2.9% | China | 2 | 0.2% |
| Finland | 27 | 2.8% | Colombia | 2 | 0.2% |
| Albania | 25 | 2.6% | Cuba | 2 | 0.2% |
| New Zealand | 24 | 2.5% | Cyprus | 2 | 0.2% |
| Sweden | 24 | 2.5% | Ethiopia | 2 | 0.2% |
| Brazil | 21 | 2.2% | Iran, Islamic Rep. | 2 | 0.2% |
| Georgia | 20 | 2.1% | Israel | 2 | 0.2% |
| Italy | 20 | 2.1% | Kazakhstan | 2 | 0.2% |
| Slovak Republic | 18 | 1.9% | Lebanon | 2 | 0.2% |
| Netherlands | 17 | 1.8% | Moldova | 2 | 0.2% |
| Romania | 17 | 1.8% | Nigeria | 2 | 0.2% |
| Czech Republic | 15 | 1.6% | Thailand | 2 | 0.2% |
| Canada | 14 | 1.5% | United Arab Emirates | 2 | 0.2% |
| Chile | 14 | 1.5% | Uruguay | 2 | 0.2% |
| Portugal | 13 | 1.4% | Algeria | 1 | 0.1% |
| Egypt, Arab Rep. | 12 | 1.3% | Bulgaria | 1 | 0.1% |
| South Africa | 12 | 1.3% | Costa Rica | 1 | 0.1% |
| Turkey | 12 | 1.3% | Dominican Republic | 1 | 0.1% |
| Lithuania | 11 | 1.2% | Indonesia | 1 | 0.1% |
| Argentina | 8 | 0.8% | Ireland | 1 | 0.1% |
| Switzerland | 8 | 0.8% | Kuwait | 1 | 0.1% |
| Austria | 7 | 0.7% | Nepal | 1 | 0.1% |
| Estonia | 6 | 0.6% | Pakistan | 1 | 0.1% |
| Poland | 6 | 0.6% | Paraguay | 1 | 0.1% |
| Korea, Rep. | 5 | 0.5% | Qatar | 1 | 0.1% |
| Singapore | 5 | 0.5% | Tunisia | 1 | 0.1% |
| Taiwan | 5 | 0.5% | Uganda | 1 | 0.1% |
| Australia | 4 | 0.4% | Vietnam | 1 | 0.1% |

Supplement Table B3. Comparison of geographic region between the 951 included and the 806 respondents excluded from the 1,757 screened healthcare professionals.

| **Region** | **Included (n = 951)** | **Excluded (n = 806)** |
| --- | --- | --- |
| Eastern Europe | 264 (27.8%) | 170 (21.1%) |
| Southern Europe | 174 (18.3%) | 251 (31.1%) |
| Western Europe | 134 (14.1%) | 61 (7.6%) |
| Northern Europe | 61 (6.4%) | 45 (5.6%) |
| Asia | 96 (10.1%) | 76 (9.4%) |
| Latin America | 84 (8.8%) | 70 (8.7%) |
| North America | 50 (5.3%) | 34 (4.2%) |
| Africa | 38 (4.0%) | 35 (4.3%) |
| Oceania | 28 (2.9%) | 33 (4.1%) |
| Middle East | 22 (2.3%) | 14 (1.7%) |
| Other / unclassified | 0 (0.0%) | 17 (2.1%) |
| **Total** | **951 (100.0%)** | **806 (100.0%)** |

Chi-square test: chi2(10) = 78.58, p = 9.5 x 10^-13. European share (any European region) 66.6% in included vs 65.4% in excluded. Country of practice was the only demographic item recorded for all 1,757 screened HCPs; sector, level of care and specialty were not available for the 502 respondents with fully missing demographic data (Supplement Figure A) and are therefore not compared here.

Supplement C

*Supplement Figure C1: Silhouette analysis – Ward Hierarchical clustering on 4 monitoring variables*

*Supplement Figure C2: Algorithm sensitivity: Ward vs k-means partitions. Cross-tabulation of cluster assignments produced by Ward agglomerative clustering (rows) and k-means clustering on the same standardised four-variable matrix (n = 951; k = 4).*

*Supplement Figure C3. Cluster profiles across candidate solutions (k = 2 to k = 6). C= Cluster*

Cluster profiles at k = 2 to k = 6 obtained by Ward agglomerative hierarchical clustering on the four standardised monitoring variables (Clinical assessment, Spirometry, FeNO, Home Monitoring Tools). At k = 5, the Clinical cluster (C2 in the k = 4 solution, n = 449) subdivides into a "Clinical assessment with minimal spirometry" subgroup (n = 237, 24.9%; spirometry mean 1.42, FeNO 0.73) and a "Clinical assessment with moderate spirometry but near-zero FeNO" subgroup (n = 212, 22.3%; spirometry mean 3.14, FeNO 0.08). At k = 6, the Low-intensity cluster (C1 in the k = 4 solution, n = 88) further divides into an extremely small (n = 5, 0.5%) minimal-monitoring subgroup with means below 1.4 on every variable, and a moderately low-intensity subgroup (n = 83, 8.7%). Both subdivisions represent intensity gradients within existing profiles rather than qualitatively new monitoring approaches, and the k = 6 minimal-monitoring subgroup is too small to be clinically actionable. Non-parametric bootstrap resampling yielded mean Jaccard similarities of 0.62 for the Low-intensity cluster (reasonably stable), 0.45 for the Clinical cluster, 0.45 for the Clinical & Objective cluster, and 0.37 for the Comprehensive cluster, indicating reasonable stability of the extreme Low-intensity profile and modest stability of the intermediate profiles, consistent with the uniformly low silhouette values.

| **Region** | **C1 (Low-intensity)** | **C2 (Clinical)** | **C3 (Clinical & Objective)** | **C4 (Comprehensive)** | **Total (n)** |
| --- | --- | --- | --- | --- | --- |
| Western Europe | 6 (4.5%) | 44 (32.8%) | 75 (56.0%) | 9 (6.7%) | 134 |
| Northern Europe | 8 (13.1%) | 20 (32.8%) | 30 (49.2%) | 3 (4.9%) | 61 |
| Southern Europe | 13 (7.5%) | 70 (40.2%) | 66 (37.9%) | 25 (14.4%) | 174 |
| Eastern Europe | 21 (8.5%) | 103 (41.9%) | 81 (32.9%) | 41 (16.7%) | 246 |
| North America | 4 (8.0%) | 24 (48.0%) | 21 (42.0%) | 1 (2.0%) | 50 |
| Latin America | 4 (4.8%) | 68 (81.0%) | 5 (6.0%) | 7 (8.3%) | 84 |
| Asia | 15 (15.6%) | 53 (55.2%) | 14 (14.6%) | 14 (14.6%) | 96 |
| Middle East | 1 (4.5%) | 14 (63.6%) | 5 (22.7%) | 2 (9.1%) | 22 |
| Oceania | 5 (17.9%) | 18 (64.3%) | 3 (10.7%) | 2 (7.1%) | 28 |
| Africa | 8 (21.1%) | 27 (71.1%) | 1 (2.6%) | 2 (5.3%) | 38 |
| **Overall** | **85 (9.1%)** | **441 (47.3%)** | **301 (32.3%)** | **106 (11.4%)** | **933** |

*Supplement Table C1. Region-by-cluster contingency table for the analytical cohort (n = 933 HCPs with complete data). Cell entries are number of respondents and (row percentage). Cluster labels: C1 = Low-intensity; C2 = Clinical (reference); C3 = Clinical & Objective; C4 = Comprehensive.*

*Supplement Table C2. Gower-distance sensitivity analysis.*

*Table C2a - Partition metrics*

| Approach | Sizes (C1 / C2 / C3 / C4) | Silhouette | ARI vs primary | Best-match agreement |
| --- | --- | --- | --- | --- |
| Ward / Euclidean (primary) | 88 / 449 / 308 / 106 | 0.278 |  |  |
| Ward / Gower | 261 / 220 / 264 / 206 | 0.333 | 0.233 | 44.8% |
| PAM / Gower | 310 / 141 / 309 / 191 | 0.31 | 0.266 | 52.2% |

| Approach | Cluster | n | % | History | Spirometry | FeNO | Home |
| --- | --- | --- | --- | --- | --- | --- | --- |
| Ward / Euclidean (primary) | C1 | 88 | 9.3% | 2,7 | 2,25 | 0,86 | 0,84 |
| Ward / Euclidean (primary) | C2 | 449 | 47.2% | 4 | 2,23 | 0,42 | 0,67 |
| Ward / Euclidean (primary) | C3 | 308 | 32.4% | 4 | 3,54 | 2,53 | 0,56 |
| Ward / Euclidean (primary) | C4 | 106 | 11.1% | 4 | 2,85 | 2,57 | 2,61 |
| Ward / Gower | C1 | 261 | 27.4% | 3,74 | 1,39 | 0,13 | 0,65 |
| Ward / Gower | C2 | 220 | 23.1% | 3,9 | 3,52 | 0,28 | 0,72 |
| Ward / Gower | C3 | 264 | 27.8% | 3,93 | 2,79 | 2,01 | 0,95 |
| Ward / Gower | C4 | 206 | 21.7% | 3,97 | 3,5 | 3,34 | 1,19 |
| PAM / Gower | C1 | 310 | 32.6% | 3,79 | 1,63 | 0,12 | 0,57 |
| PAM / Gower | C2 | 141 | 14.8% | 3,87 | 3,8 | 0,17 | 0,98 |
| PAM / Gower | C3 | 309 | 32.5% | 3,93 | 2,76 | 2,07 | 0,73 |
| PAM / Gower | C4 | 191 | 20.1% | 3,95 | 3,65 | 3,22 | 1,48 |

*Table C2b - Cluster means*

Supplement Table D. All monitoring parameters in PeARL survey

| **Monitoring item** | **Mean C1 (n=88)** | **Mean C2 (n=449)** | **Mean C3 (n=308)** | **Mean C4 (n=106)** | **Overall mean** | **Δmean (max−min)** | **Within-cluster SD (pooled)** | **Between/within ratio** | **KW H** | **KW p** | **ε² (effect size)** |
| --- | --- | --- | --- | --- | --- | --- | --- | --- | --- | --- | --- |
| Peak flow rate | 1.64 | 1.76 | 1.67 | 2.55 | 1.8 | **0.91** | 1.41 | 0.65 | 32.7 | **< 0.001** | **0.03** |
| Skin-prick tests | 1.25 | 1.52 | 1.79 | 1.84 | 1.62 | **0.59** | 0.84 | 0.7 | 40.1 | **< 0.001** | **0.04** |
| Growth monitoring | 2.83 | 3.34 | 3.59 | 3.37 | 3.38 | **0.76** | 0.92 | 0.83 | 47.6 | **< 0.001** | **0.04** |
| General adherence history | 3 | 3.57 | 3.75 | 3.68 | 3.59 | **0.75** | 0.8 | 0.94 | 62.4 | **< 0.001** | **0.06** |
| Inhalation-technique assessment | 2.92 | 3.3 | 3.4 | 3.28 | 3.29 | **0.48** | 0.83 | 0.57 | 15.9 | **<0.001** | **0.01** |
| In-clinic PROs (composite of ACT, ACQ and CASI) | 1.52 | 1.46 | 1.57 | 2.1 | 1.57 | **0.64** | 0.94 | 0.68 | 43.9 | **< 0.001** | **0.04** |

*Δmean ≥ 2.0: very strong discrimination; 1.0–2.0: strong discrimination; 0.5–1.0: weak discrimination; < 0.5 no discrimination,*

*ε² < 0.01: negligible; 0.01–0.06: small; 0.06–0.14: medium; ≥ 0.14: large*
